# Home Department

**Published:** 1886-05

**Authors:** 


### HOME DEPARTMENT.

**To get rid of Roaches.**—Take the thick peeling of cucumbers, strew them on the floor and leave them there for the night. In the morning there will be some thousands of dead roaches, if the premises contain so many. If not, repeat the dose for two or three nights and you will have no roaches on your premises.

**Corn Bread.**—Corn bread made of the granulated or the "new process" corn meal, is wholesome for breakfast. Mix one cup of yellow meal, and two cups of flour with two teaspoonfuls of baking powder, and one of salt. Stir into this one tablespoonful of butter or nice drippings and one small cup of sugar; then add the beaten yolks of two eggs and enough milk to make a thin batter. When mixed, add the beaten whites of the two eggs. Bake fifty minutes in a moderate oven. Be careful to have a thin batter.

**Maccaroni.**—Maccaroni is perhaps the most nutritious of all preparations of wheat. It owes its superior properties to the hardness of the grain selected for its manufacture. The best of wheat for the purpose is the excessively hard variety grown in Egypt and the northern parts of Africa. It is the staple food of the southern parts of Italy, but is gradually extending all over Europe, having also received much impetus from the International Exhibition of 1872, where the process of its manufacture and the mode of its preparation were both exhibited.

**For Unfermented Bread.**—The following recipe will be found advantageous: Three pounds wheat meal; half an ounce (avoirdupois) muriatic acid; half an ounce carbonate of soda, water enough to make it of a proper consistence.

*For White Four*—four pounds of flour; half an ounce muriatic acid; half an ounce carbonate soda; water about a quart. The way of making is as follows: First mix the soda and flour well together by rubbing in a pan; then pour the acid into the water, and mix well by stirring. Mix all together to the required consistence and bake in a hot oven immediately. The gain from this method of baking is as follows: four pounds of wheat meal make seven pounds nine ounces of excellent light bread; and four pounds of white flour make six pounds of excellent light bread. It keeps moist longer than bread made with yeast, and is far more sweet and digestible; this is especially recommended to persons who suffer from indigestion, who will find the brown bread invaluable.

**Onions**.—Almost every one has either a special liking or a special dislike of onions. In addition to the peculiar flavor for which they are sought, they are remarkably nutritious. The growth of onions requires a rich soil, and favorable rains and a hot sun; hence the great superiority of the Spanish onion.

**Browned Sweet Potatoes**.—Take sweet potatoes left over from a previous meal, if they have not been pealed, remove the skin, then mash fine, spread on a pie pan, and leave in a brick oven from fifteen to twenty minutes. They will be as good as the freshly cooked potatoes. Another way equally as good is to slice instead of mashing them.

**Potatoes**.—Potatoes are more important as a variety of food than any other root, being grown over a greater range of latitude than any other cultivated plant. The dry substance they contain, known as potatoe meal, is unsuited for bread alone, though it is extensively used as an adulturment of wheaten flour, and is said to improve the bread in some respects, though it renders it inferior in others. This will be appreciated when it is considered that potato flour, weight for weight, is less nutritive in the sense of supplying strength and endurance than any other meal except that of rice and the plantain.

**Rice**.—Rice is is remarkable chiefly for the comparatively small proportion of gluten it contains, not exceeding seven or eight per cent., being less than half the quantity contained in oat meal. When substituted for potatoes in work-houses, it has been observed to produce scurvy. Upon the whole, rice contains less nutriment than any other grain, being also considerably inferior to the potato. The natives of India make up for the deficiency of relative nutriment by devouring enormous quantities, for which their large stomachs are adapted; but even with that advantage it is very inferior food, and results in an inferior population.

**Breakfast Rolls**.—For light rolls take one quart of flour one spoonful of clean beef drippings one teaspoonful of salt, two teaspoonfuls Royal Baking Powder. With the hands rub all these ingredients smoothly together, then with a spoon mix up with milk to a consistency to roll out, as soft as can well be handled, and handle as little and as lightly as possible. Roll to about one inch in thickness, cut out with a tumbler, lay on a bit of butter and fold over once, evenly. Bake in a quick oven about half an hour. These rolls are good hot, cold or steamed. The same rule will make good light biscuit by rolling thicker and cutting in plain round form.

**Hot Water Rolls**.—The various uses of "Grahan" Flour are quite generally known, but the use of that flour in every family is so important that this subject cannot be overdone. These rolls are sweet and wholesome, especially if the dough is mixed pretty stiff and the bread thoroughly baked. They are made by pouring boiling water into a quantity of unsifted Graham flour, stirring constantly with a strong iron spoon until two-thirds of it is scalded; then finish with cold water, stirring with the spoon making the dough stiff enough to handle with the hands; if too stiff, the bread will not be good, then pinch off in small bits and make into rolls an inch thick and about three inches in length; form by rolling on the moulding-board, sprinkled with dry flour to prevent sticking. Put them into a bread-pan, spacing so that they will not touch each other and bake thirty or fourty minutes in a very hot oven. This is good warm bread, but it is also good cold.

### RECENT PUBLICATIONS.

**Vick's Illustrated Monthly**—Is at hand. It come with the first flowers of May and is as interesting to all who appreciate that ornamental part of nature's work. Everybody who cultivates flowers should have the magazine which cost but $1.25 for a year.

**The Century**—For May, is full of the very choicest reading. It continues its war papers which interest so many of our old soldiers and their friends. It is embellished with a fine portrait of Nathaniel Hawthorne. Its article on "American Country Dwellings," is valuable. In fact, we can only repeat that the Century's articles are good for all, for it is so conducted, that it has something for all classes who read.

**The Phrenological Journal**—Comes to us, as of old, full of most excellent reading, instructing and entertaining. "The Disciples of Christ," is an article that will attract attention from those known by that name. It has an excellent portrait of Rev. Robert Graham with a sketch of his life. "The Constitutional Basis of Character," is an able and interesting paper. "Backs and Characters" is a gem, a grand article to read and study, and also to amuse. In fact the whole is excellent. Twenty cents will purchase the copy while $2 will get it for a year and will be money well expended.

